# Cellular proteostasis decline in human senescence

**DOI:** 10.1038/s42003-020-01578-w

**Published:** 2021-01-04

**Authors:** Karli Montague-Cardoso

**Affiliations:** Communications Biology, https://www.nature.com/commsbio

## Abstract

A huge amount of intrigue surrounds the aging process. Senescence—the decreased likelihood of reproduction and the increased chance of mortality—is a hallmark of aging. The reduced ability of senescent cells to maintain protein homeostasis (proteostasis) has been well-established in nematodes but this phenomenon had yet to be directly demonstrated in human cells. Sabath et al. recently provided compelling evidence that proteostasis collapse is indeed intrinsic to human cell senescence, which may have broad implications in the underlying processes of human aging.

Aging is associated with a diminished ability of cells to respond to external stressors as well as the accumulation of damaged proteins. Whilst we are in possession of extensive evidence that indicates disruption of proteostasis is a hallmark of senescence in nematodes, direct evidence for disruption to protein homeostasis and quality control in human cells has yet to be obtained. Examining senescent human cell responses to stress and associated changes in protein handling constitutes an important step in beginning to elucidate the potential role of proteostasis disruption in senescence in humans.

**Figure:**
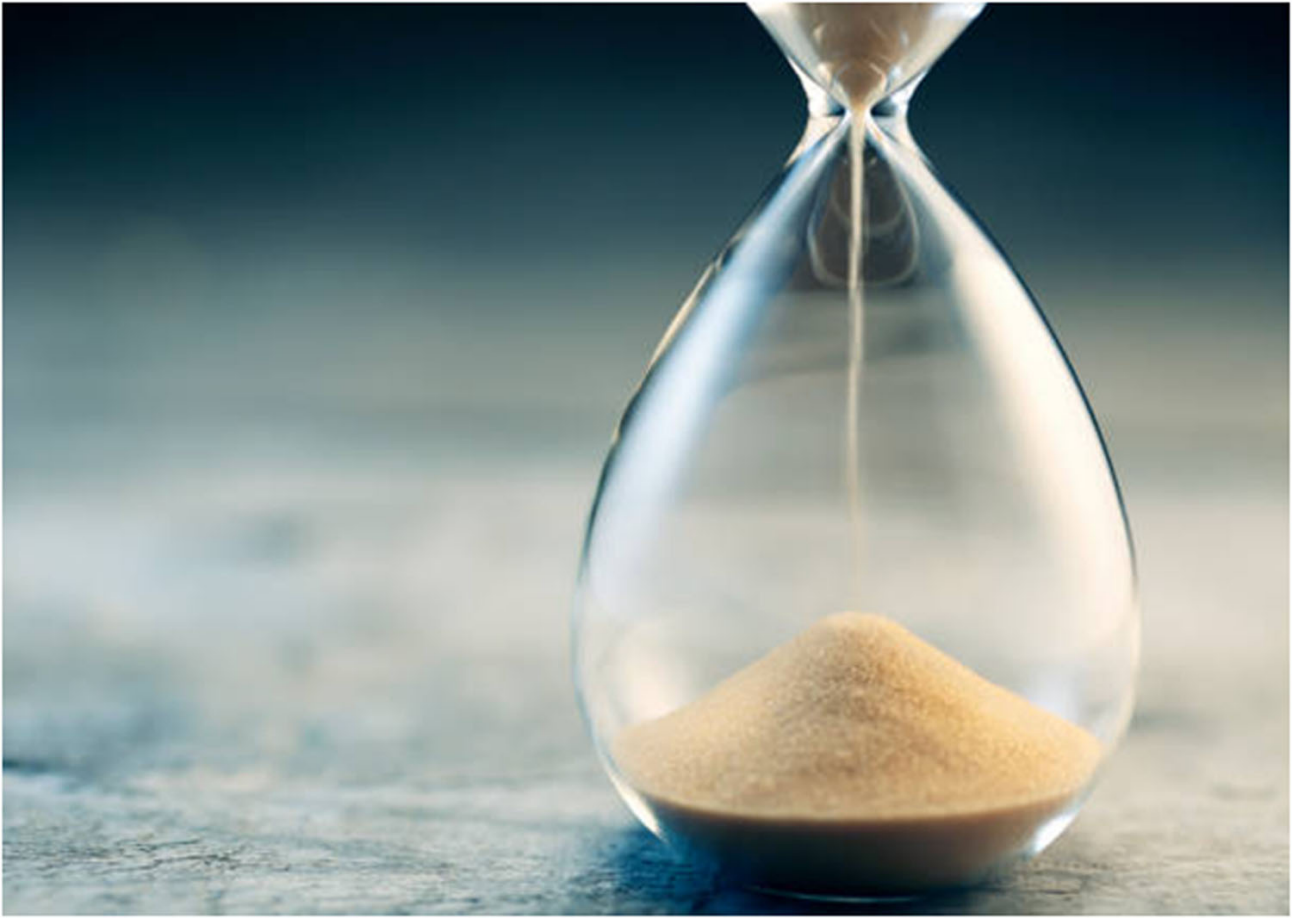
Pixabay.

Sabath et al.^1^ recently used transcriptome-wide characterization of gene expression, splicing and protein translation in primary human fibroblast cells, which were exposed to heat stress. The cells were either young or cultured until considered senescent. They used RNA-seq and subsequent validation to provide compelling evidence for a significant deterioration of transcriptional activation of the heat shock response in senescent cells. In addition, using immunofluorescence, they observed that the nuclear localisation and distribution of phosphorylated/activated Heat Shock Factor 1 were impaired, also indicative of a disruption in the response of senescent cells to heat shock. Intriguingly, they also observed that proteostasis disruption was evident at the level of alternative splicing.

When cells are stressed, the endoplasmic reticulum (ER) responds by activating the unfolded protein response (UPR), which consists of three signalling branches. Surprisingly, Sabath et al. also found evidence for decoupling between these different branches in stressed senescent cells. They observed that whilst younger cells were able to initiate UPR-related transcriptional and translational responses as expected, older, senescent cells were unable to trigger UPR-related transcriptional responses despite showing a heightened translational regulation and ER stress sensing. The inability of the senescent cells to elicit UPR-related transcriptional changes was also accompanied by reduced nuclear localisation of ATF6, which mediates one of the branches of the UPR. The changes observed with respect to both ATF6 and HSF1 nuclear localisation are in support of the notion of compromised nuclear integrity in aging. Finally, they also showed that the impaired stress response of heat-stressed senescent cells was not restored when a normal temperature was restored.

Taken together this study presents convincing evidence that senescent cells possess a deteriorated ability to regulate stress-related transcriptional programs and as such proteostasis is disrupted in a cell autonomous manner. Sabath et al. make an important advance in building upon our understanding of the mechanisms underlying senescence in human aging.

## References

[1] Sabath N (2020). Cellular proteostasis decline in human senescence. Proc. Natl Acad. Sci. USA.

